# Genomic characterization of carbapenem-resistant organisms from pediatric patients with hematological malignancies and genomic comparison of infection versus intestinal colonizing strains

**DOI:** 10.1128/spectrum.01719-26

**Published:** 2026-10-02

**Authors:** Chao Fang, Zheng Zhou, Jianping Li, Xiucai Zhang, Jintao Xia, Shixing Liu, Yining Zhao, Xiaojun Xu, Shiqiang Shang, Mingming Zhou

**Affiliations:** 1 Department of Clinical Laboratory, Children’s Hospital, Zhejiang University School of Medicine, National Clinical Research Center for Children and Adolescents' Health and Diseases 605254, Hangzhou, Zhejiang, China; 2 Department of Hematology, Children’s Hospital, Zhejiang University School of Medicine, National Clinical Research Center for Children and Adolescents' Health and Diseases 605254, Hangzhou, Zhejiang, China; London Health Sciences Centre, London, Ontario, Canada

**Keywords:** carbapenem-resistant organisms, genomics, pediatrics, hematological malignancies, infections, colonization

## Abstract

This study aimed to characterize the species distribution, sequence types, and antimicrobial resistance mechanisms of carbapenem-resistant organisms (CROs) in pediatric patients with hematological malignancies, and to clarify the genomic relationship between clinical infectious CROs and intestinal colonizing CROs. Non-duplicate infectious CRO strains and paired intestinal colonizing CROs were collected from 2020 to 2024. Whole-genome sequencing and bioinformatics analyses were performed to identify bacterial species, multilocus sequence types, resistance genes, mobile genetic elements, and genomic relatedness. Seven CRO species were identified, dominated by *Pseudomonas aeruginosa*, *Klebsiella pneumoniae*, and *Enterobacter hormaechei*. A total of 34 carbapenem resistance-associated genes were detected, with efflux pump overexpression and antibiotic inactivation as the main mechanisms. Three patients carried both infectious and intestinal CROs. Two pairs showed nearly identical genomes (average nucleotide identity >99.99%), confirming infections originated from intestinal colonization. One pair belonged to different species but shared an identical *KPC-2* gene located on the same Tn*7247*-like transposon, indicating potential horizontal gene transfer. Diverse CROs and corresponding resistance mechanisms are prevalent in pediatric patients with hematological malignancies. This study identifies the dominant CRO species and key resistance mechanisms in this high-risk population, supporting the gastrointestinal tract as a potential reservoir for CROs, and indicating the likelihood of horizontal transmission of carbapenemase genes. These findings underscore the necessity of intensive surveillance and strict infection control measures to curb the dissemination of CROs in vulnerable pediatric populations.

**IMPORTANCE:**

Carbapenem-resistant organisms (CROs) cause life-threatening infections in pediatric patients with hematological malignancies, who are highly vulnerable due to chemotherapy-induced immunosuppression. This study characterized CRO species, resistance mechanisms, and genomic links between infectious and intestinal colonizing strains in this population. We confirmed that intestinal colonization is an important source of subsequent CRO infections and identified interspecies horizontal transfer of the carbapenemase gene *KPC-2* via a Tn*7247*-like transposon. These findings highlight the critical need for enhanced intestinal CRO screening and strict infection control measures to protect this vulnerable pediatric group.

## INTRODUCTION

The global emergence and spread of carbapenem-resistant organisms (CROs) have become a severe threat to public health and clinical antimicrobial therapy, especially for immunocompromised patient populations (1, 2). Pediatric patients with hematological malignancies are particularly vulnerable to life-threatening CRO infections due to intensive chemotherapy, prolonged neutropenia, impaired mucosal immunity, and frequent exposure to broad-spectrum antibiotics (3–5). Invasive infections caused by CROs in this population are associated with high rates of treatment failure, prolonged hospital stays, and increased mortality (6, 7).

The gastrointestinal tract is widely recognized as a major reservoir for CRO colonization, which can serve as the source for subsequent endogenous infections, including bloodstream, respiratory, and surgical site infections (8–10). However, the genomic relationship between intestinal colonizing CROs and clinical infectious CRO isolates remains poorly characterized in pediatric patients with hematological malignancies. At the molecular level, carbapenem resistance in gram-negative bacteria is mainly mediated by carbapenemase production, overexpression of efflux pumps, decreased outer membrane permeability, and antibiotic target modification (11–13). Among these mechanisms, carbapenemase genes are frequently located on plasmids, transposons, and other mobile genetic elements, enabling rapid horizontal gene transfer across species and genera (14–16). Despite extensive studies in adult populations, data regarding the distribution of resistance genes and molecular mechanisms of CROs specifically in pediatric hematology settings remain limited. Moreover, most previous studies have focused on either colonizing or infectious CRO isolates alone, and few studies have systematically compared their genomic relatedness, resistance gene profiles, and genetic contexts (17–19). Clarifying whether clinical infectious CRO strains originate from intestinal colonization is critical for optimizing infection control, antimicrobial stewardship, and targeted surveillance (20–22).

Therefore, the present study was conducted to characterize the species distribution, multilocus sequence types (STs), antimicrobial resistance (AMR) gene profiles, and carbapenem resistance mechanisms of CROs isolated from pediatric patients with hematological malignancies. Furthermore, we conducted comparative genomic analysis between intestinal colonizing CROs and their corresponding infectious isolates to explore their genetic relatedness and potential transmission routes. This study provides valuable genomic insights into the origin and dissemination of CROs in this high-risk pediatric population, with implications for clinical prevention and control strategies.

## MATERIALS AND METHODS

### Strains

The CRO infectious strains included in this study were non-duplicate isolates recovered from clinical infectious specimens of pediatric patients with hematological malignancies hospitalized at the Children’s Hospital, Zhejiang University School of Medicine between 2020 and 2024. Initially, preliminary screening and identification of the bacterial isolates were performed using matrix-assisted laser desorption–ionization time-of-flight mass spectrometry (Bruker Daltonik, Bremen, Germany) and the Vitek 2 Compact system (bioMérieux, Marcy-l’Étoile, France). Susceptibility of each isolate to imipenem (IPM, Oxoid Limited, Basingstoke, United Kingdom), meropenem (MEM, Oxoid Limited, Basingstoke, United Kingdom), and ertapenem (ETP, Oxoid Limited, Basingstoke, United Kingdom) was subsequently verified by the Kirby-Bauer (K-B) disk diffusion method. Relevant clinical and demographic data, including patient age, gender, specimen source, and clinical diagnosis, were extracted from electronic medical records and standardized laboratory databases. Subsequent systematic collation and verification were performed to ensure the accuracy and consistency of the data.

Intestinal colonizing CRO isolates were derived from routine screening for CRO conducted among pediatric patients with hematological malignancies at our hospital. The screening protocol was performed as follows: rectal swab specimens were collected from children with hematological malignancies hospitalized for more than 48 hours, inoculated onto MacConkey agar plates (Beruite Biotechnology Co., Ltd, Zhengzhou, China), and supplemented with a meropenem disk, followed by incubation at 35°C for 48 hours. Bacterial growth observed at or within the resistance breakpoint zone was regarded as a suspected CRO isolate and subsequently purified for further confirmation. Antimicrobial susceptibility confirmation was performed using both the Vitek 2 Compact system and the K-B method (for verification of IPM, MEM, and ETP). Only isolates confirmed to be carbapenem-resistant by both methods were defined as intestinal colonizing CRO strains. In addition, repeat screening for intestinal CRO colonization was performed by clinicians based on the length of hospital stay, antibiotic exposure, and chemotherapy status of pediatric patients with hematological malignancies; accordingly, the timing of isolation of intestinal colonizing CRO isolates was not fixed. In the present study, only intestinal colonizing CRO strains recovered within a 2-week window before or after isolation of the corresponding infectious CRO isolate were included for comparative genomic analysis. Antimicrobial susceptibility profiles were interpreted according to the clinical breakpoints published in the Clinical and Laboratory Standards Institute (CLSI) M100-S35 guidelines (23). Since some strains exhibit intrinsic resistance to certain carbapenems, we adopted the following definitions in this study: During screening for CRO, strains that were resistant only to carbapenems to which they show intrinsic resistance but susceptible to other carbapenems were not classified as CRO and were excluded from the study. In antimicrobial susceptibility testing, if an isolate exhibits intrinsic resistance to any carbapenem antibiotic, the susceptibility of that carbapenem agent against the strain is defined as resistant regardless of the results of phenotypic susceptibility testing. *Escherichia coli* (*E. coli*) ATCC 25922 and *Pseudomonas aeruginosa* (*P. aeruginosa*) ATCC 27853 were used as quality control strains throughout all experimental procedures.

### DNA extraction and sequencing

Genomic DNA was isolated from each bacterial strain using the FastPure Bacteria DNA Isolation Mini Kit (Vazyme Biotech Co., Ltd., Nanjing, China) in strict compliance with the manufacturer’s recommended protocols. The purity (evaluated by A260/A280 absorbance ratios) and concentration of the extracted genomic DNA were quantified using a BioDrop mLite+ spectrophotometer (BioDrop, Cambridge, UK) to ensure the samples met the quality criteria for subsequent whole-genome sequencing (WGS) experiments. WGS was carried out on an Illumina HiSeq X Ten sequencing platform (Illumina, San Diego, CA, USA) adopting a paired-end sequencing strategy. Raw sequencing reads were subjected to quality filtering using fastp v.0.23.2 (24), with the following criteria for exclusion: reads containing more than 5% ambiguous bases (N), more than 50% low-quality bases (Phred quality score Q ≤ 5), adapter contamination, or PCR duplicates. *De novo* assembly of the quality-filtered reads into contigs was performed using the Unicycler bioinformatics pipeline (v.0.5.0) (25), a tool specialized for high-accuracy bacterial genome assembly. Genome completeness and potential contamination were assessed using CheckM2 v.1.1.0 (26), with strict thresholds set as follows: genome completeness ≥99.0% and contamination ≤0.5%.

### WGS analysis

Gene annotation of all genomic sequences was performed using the Prokka v.1.14.6 pipeline (27), a widely used tool for rapid and accurate prokaryotic genome annotation. For multilocus sequence typing (MLST) analysis of CRO strains (encompassing multiple bacterial species rather than a single species), searches were conducted against the corresponding species-specific MLST databases hosted on the PubMLST platform. AMR genes were predicted and annotated using the Resistance Gene Identifier tool integrated within the Comprehensive Antibiotic Resistance Database (CARD) v.4.0.1 (https://card.mcmaster.ca/analyze/rgi), with all analyses performed under the platform’s default detection thresholds, as well as CARD-curated bitscore cut-offs. Average nucleotide identity (ANI) was determined between the intestinal colonizing isolate and the infecting isolate using FastANI v.1.34 (28). Pairwise genome comparison was performed with the command fastANI -q query.fasta -r reference.fasta -o result.ani, where -q and -r indicated the genome sequences of the colonizing isolate and infecting isolate, respectively, and -o specified the output file. Isolates with an ANI value of ≥99.99% were considered to belong to the same strain, while those with ANI ≥95% were classified as the same species (28, 29). Insertion sequences (ISs) within the genomic sequences were screened using ISfinder (30), a dedicated database and analysis tool for the identification of prokaryotic insertion sequences. Transposons were retrieved and annotated via TnCentral (https://tncentral.ncc.unesp.br/), a comprehensive annotated database of prokaryotic transposable elements. Heat maps were constructed using the “pheatmap” package (v.1.0.12) (31) in R software (v.4.2.2) through Hiplot Pro (https://hiplot.com.cn/). Circular genome maps were generated using Proksee (https://proksee.ca/), an expert web-based system designed specifically for in-depth characterization and interactive visualization of prokaryotic genomes. Impact plots were constructed using OriginPro 2021 (OriginLab Corporation, Northampton, MA, USA), a professional data visualization and statistical analysis software widely employed in biomedical and life science research for high-quality scientific graphing and data interpretation.

## RESULTS

### General characteristics of CRO strains and study population

**Fig 1:**
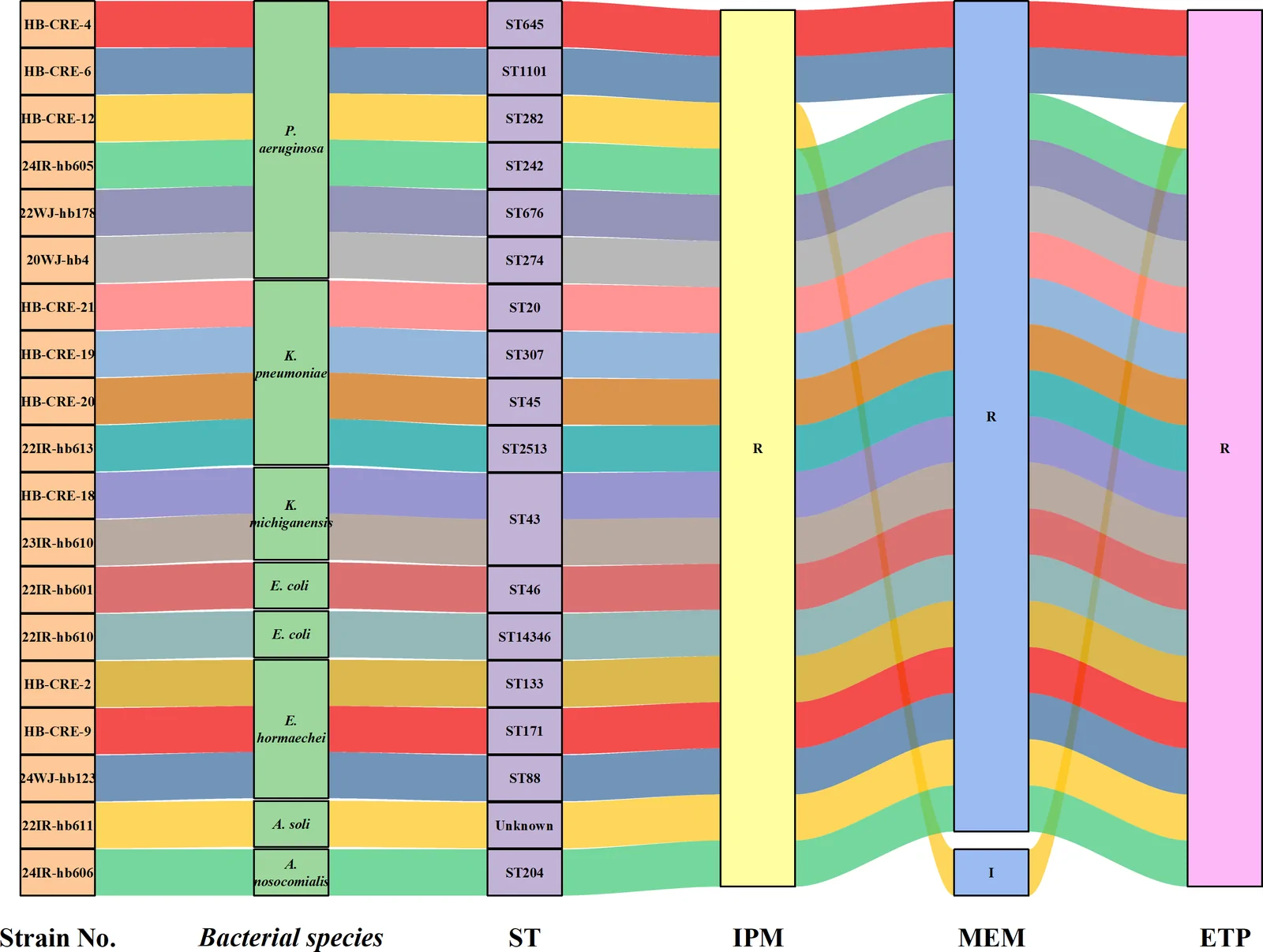
The distribution of bacterial species, STs, and susceptibility to major carbapenem antibiotics of the 19 CRO strains. The figure depicts the genomic and phenotypic characteristics of 19 non-repetitive CRO isolates. The leftmost column indicates strain identifiers. The bacterial species column shows the taxonomic classification, revealing seven distinct gram-negative species, with *P. aeruginosa*, *K. pneumoniae*, and *E. hormaechei* predominating. The ST column presents the corresponding STs, most of which were unique to each isolate, except for two *K. michiganensis* strains sharing ST43. The rightmost three columns represent the *in vitro* susceptibility to IPM, MEM, and ETP, respectively, interpreted according to CLSI M100-S35 guidelines. Color gradients represent the zone of inhibition diameter, with R denoting resistant and I denoting intermediate susceptibility, collectively confirming that all isolates exhibited phenotypic resistance to carbapenems.

A total of 19 non-repetitive CRO strains were isolated from infectious specimens of 19 hospitalized children with hematological malignancies. The susceptibility of each strain to clinically common carbapenem antibiotics (IPM, MEM, and ETP) was reconfirmed by the K-B method, and the antimicrobial susceptibility results are presented in Fig. 1. Demographic characteristics of the 19 children were summarized as follows: in terms of age distribution, 15.8% (3/19) were <1 year old, 36.8% (7/19) were 1 to <5 years old, and 47.4% (9/19) were ≥5 years old. Regarding gender distribution, 47.4% (9/19) were male, and 52.6% (10/19) were female. For underlying diseases, 57.9% (11/19) of the children were diagnosed with acute lymphoblastic leukemia (ALL), and 42.1% (8/19) with acute myeloid leukemia (AML). Blood was the most common source of specimens, accounting for 52.6% (10/19), while the remaining 47.4% were isolated from various specimens, including incision secretion, pus, and sputum. Species identification revealed seven different bacterial species among the 19 CRO strains. The three most frequently detected species were *P. aeruginosa* (6/19, 31.6%), *Klebsiella pneumoniae (K. pneumoniae,* 4/19, 21.1%), and *Enterobacter hormaechei (E. hormaechei,* 3/19, 15.8%). MLST showed that due to the 19 strains belonging to seven different bacterial species, all strains had distinct STs except for two *Klebsiella michiganensis* (*K. michiganensis*) strains, which both belonged to ST43. The distribution of bacterial species, STs, and susceptibility to major carbapenem antibiotics of the 19 CRO strains is shown in Fig. 1.

### Analysis of carbapenem resistance-associated genes

A total of 217 drug resistance-related genes were detected in the 19 CRO strains, among which 34 (34/217, 15.7%) were associated with carbapenem resistance. From the perspective of resistance mechanisms, these 34 carbapenem resistance-associated genes were mainly involved in antibiotic efflux (16/34, 47.1%) and antibiotic inactivation (11/34, 32.4%). Significant differences in the carriage of resistance genes and resistance mechanisms were observed among different bacterial species. The detailed distribution of carbapenem resistance-associated genes is described as follows:

#### E. coli

Both *E. coli* strains carried the *marA* gene, whose resistance mechanisms included reduced permeability to antibiotics and antibiotic efflux, as well as the *Ecol_soxS_MULT* gene with resistance mechanisms involving reduced permeability to antibiotics, antibiotic efflux, and antibiotic target alteration. Additionally, both strains harbored the *NDM-5* gene, which encodes class B metallo-β-lactamases (MBLs). Strain 22IR-hb601 also carried the *TolC* gene, which is associated with the antibiotic efflux mechanism.

#### K. pneumoniae

All four *K. pneumoniae* strains carried the *marA* gene (reduced permeability to antibiotics and antibiotic efflux), the antibiotic efflux-associated genes *Kpne_KpnH*, *Kpne_KpnG*, and *LptD*, as well as the reduced permeability-associated genes *MdtQ* and *Kpne_OmpK37*. Furthermore, these four strains carried three different carbapenemase genes: two strains harbored *NDM-5* (encoding class B MBLs), one strain carried *IMP-4* (encoding class B MBLs), and one strain carried *KPC-2* (encoding class A serine carbapenemases).

#### K. michiganensis

Both *K. michiganensis* strains carried the *marA* gene (reduced permeability to antibiotics and antibiotic efflux), the antibiotic efflux-associated genes *Kpne_KpnH*, *Kpne_KpnG*, and *LptD*, as well as the *KPC-2* gene (encoding class A serine carbapenemases).

#### E. hormaechei

All three *E. hormaechei* strains carried the *marA* gene (reduced permeability to antibiotics and antibiotic efflux). Each of the three strains harbored a different gene encoding class B MBLs, namely *IMP-26*, *IMP-4*, and *NDM-1*. In addition, strain 24WJ-hb123 also carried the *ACT-17* gene, which is associated with the antibiotic inactivation mechanism.

#### P. aeruginosa

The main resistance mechanism of the six *P. aeruginosa* strains was antibiotic efflux. All six strains carried the MexPQ-OpmE multidrug efflux complex, the MexAB-OprM efflux complex, the MexXY-OprM efflux complex, and the two-component sensor ParRS. Additionally, these six strains carried four different antibiotic inactivation-associated genes: *PDC-3*, *PDC-24*, *PDC-23*, and *PDC-120*. It is worth noting that although all six strains carried the *OprM*, *mexY*, and *MexZ* genes, they lacked the *mexX* gene, thus failing to form a complete MexXY-OprM efflux complex.

#### *Acinetobacter baumannii* complex (*A. baumannii complex*)

Both strains of the *A. baumannii complex* carried the *NDM-1* gene (encoding class B MBLs). In addition, strain 22IR-hb611 also harbored the *OXA-58* gene, which encodes OXA-type serine carbapenemases.

**Fig 2:**
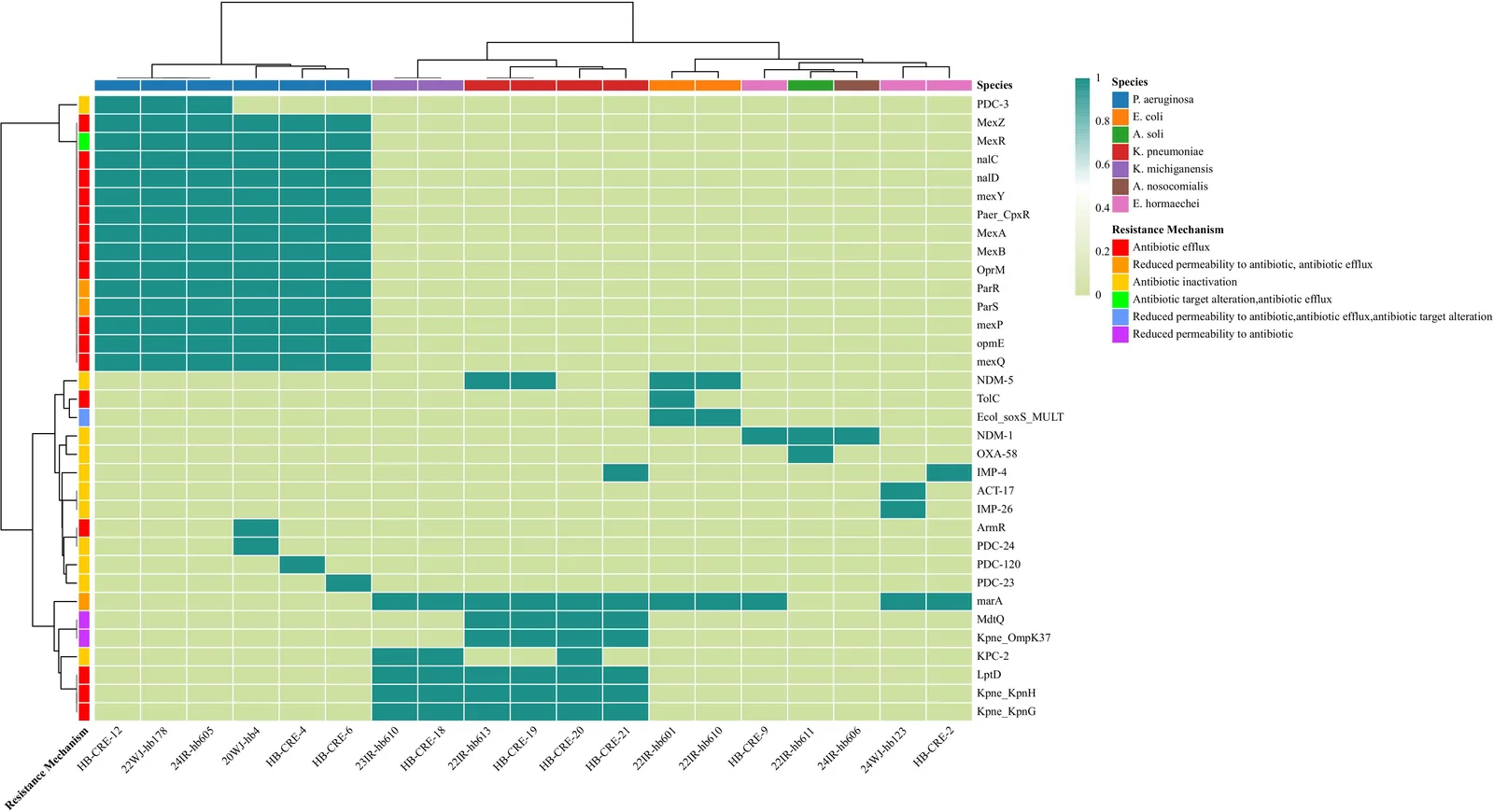
Heatmap of carbapenem resistance-associated genes in 19 CRO isolates from pediatric patients with hematological malignancies. The heatmap displays the distribution of carbapenem resistance-associated genes across 19 CRO isolates, clustered by strain and gene. Color intensity indicates gene presence, with side bars annotating bacterial species and resistance mechanisms (efflux, inactivation, permeability reduction, target alteration). The figure highlights species-specific resistance gene profiles and dominant carbapenem resistance mechanisms in this high-risk pediatric population.

The distribution of carbapenem resistance-associated genes in the 19 CRO strains is shown in Fig. 2.

### Comparative analysis of infectious strains and intestinal colonizing strains

Routine screening for intestinal CRO strains was performed in the 19 hospitalized children with hematological malignancies, and CRO strains were isolated from the intestines of three children. Comparative genomic analysis was conducted between these three intestinal colonizing strains and their corresponding infectious strains, and the results are as follows:

#### *HB-CRE-9* vs *21IR1062*

**Fig 3:**
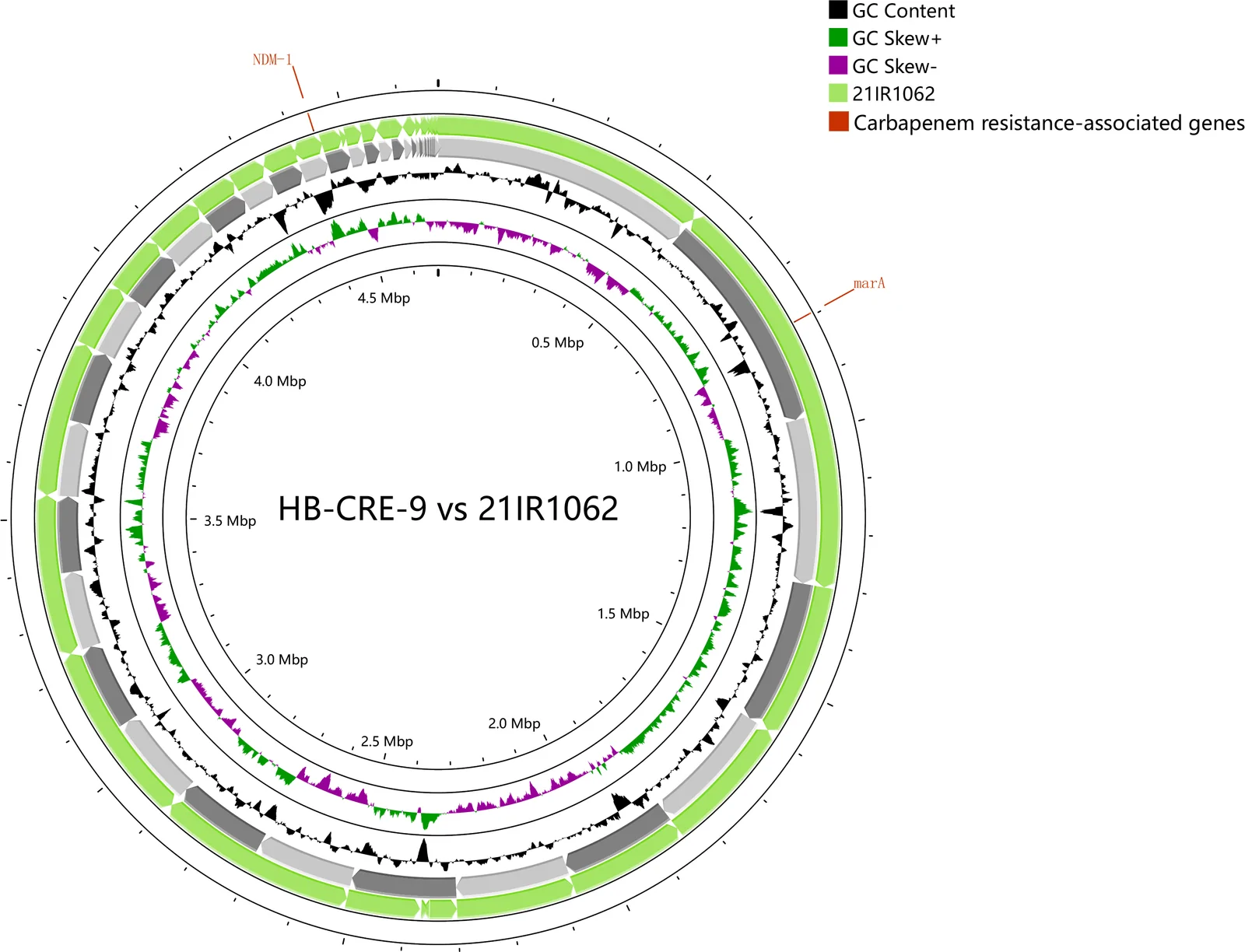
Circular genome comparison of *E. hormaechei* strains HB-CRE-9 (infectious isolate) and 21IR1062 (intestinal colonizing isolate). From innermost to outermost: the gray-white alternating ring represents the reference genome of strain HB-CRE-9; the light green ring denotes the alignment coverage of strain 21IR1062, with high identity across the entire genome. Inner tracks show GC content (black), GC skew+ (green), and GC skew- (purple). Key carbapenem resistance genes are annotated, confirming identical genetic profiles between the two strains.

Strain HB-CRE-9 was isolated from the upper respiratory tract specimen of a child and was identified as the pathogen causing upper respiratory tract infection in the patient. KmerFinder 3.2 analysis confirmed that HB-CRE-9 was *E. hormaechei* with ST171. Strain 21IR1062 was an intestinal colonizing strain that was isolated, which was also identified as *E. hormaechei* with ST171 by KmerFinder 3.2. The two strains carried identical carbapenem resistance-associated genes, namely *marA* and *NDM-1*. The ANI between the two strains was 99.998%. Comparative genomic analysis of HB-CRE-9 and 21IR1062 is shown in Fig. 3.

#### *HB-CRE-18* vs *21IR1172*

**Fig 4:**
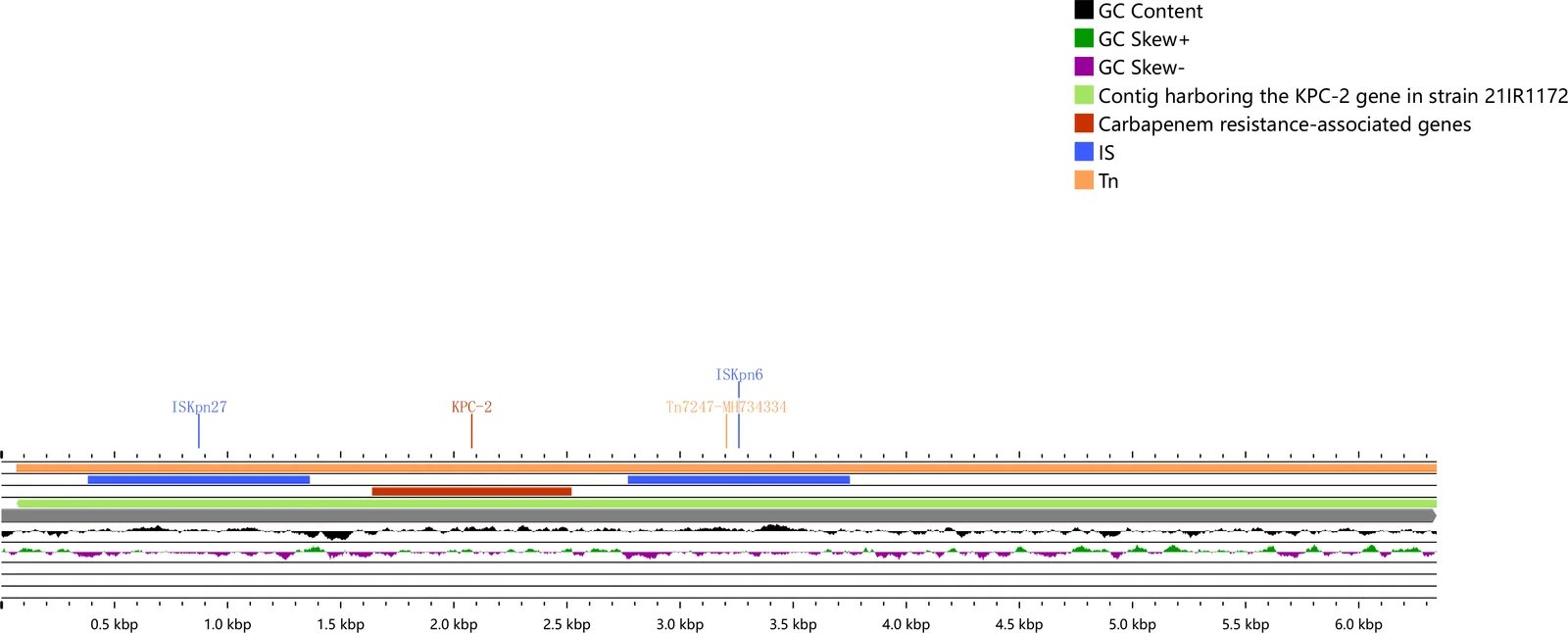
Genetic context analysis of the carbapenemase-encoding gene *KPC-2* in strains HB-CRE-18 and 21IR1172. The figure depicts the genetic environment of the *KPC-2* gene. The gray bar represents the contig sequence harboring the *KPC-2* gene in strain HB-CRE-18. The light green bar indicates the alignment coverage of the corresponding contig from strain 21IR1172, confirming high sequence identity. Tracks display GC content (black), GC skew (green/purple), insertion sequences (IS, blue), and transposons (Tn, light red). The *KPC-2* gene is flanked by IS*Kpn6* and IS*Kpn27* within a Tn*7247*-like transposon.

Strain HB-CRE-18 was isolated from the pus specimen of a child and was identified as *K. michiganensis* with ST43 by KmerFinder 3.2. Strain 21IR1172 was an intestinal colonizing strain, which was identified as *Klebsiella quasipneumoniae (K. quasipneumoniae)* with ST4627 by KmerFinder 3.2. Both strains carried the carbapenem resistance-associated genes *marA*, *Kpne_KpnH*, *Kpne_KpnG*, *LptD*, and *KPC-2*. However, the carbapenem resistance-associated genes *Kpne_OmpK37* and *MdtQ* were only detected in 21IR1172 but not in HB-CRE-18. The ANI between the two strains was 85.1778%. In addition, we compared the genetic background of the contig harboring the mobile resistance gene *KPC-2* in the two strains. The *KPC-2* sequences carried by the two strains were completely identical without mutations. Meanwhile, the genetic background of the contig harboring the *KPC-2* was also identical in both strains: the *KPC-2* was located within a Tn*7247*-like transposon, with insertion sequences IS*Kpn6* and IS*Kpn27* flanking the *KPC-2*. The analysis of the genetic background of the *KPC-2* carried by HB-CRE-18 and 21IR1172 is shown in Fig. 4.

#### *22WJ-hb178* vs *22IR1128*

**Fig 5:**
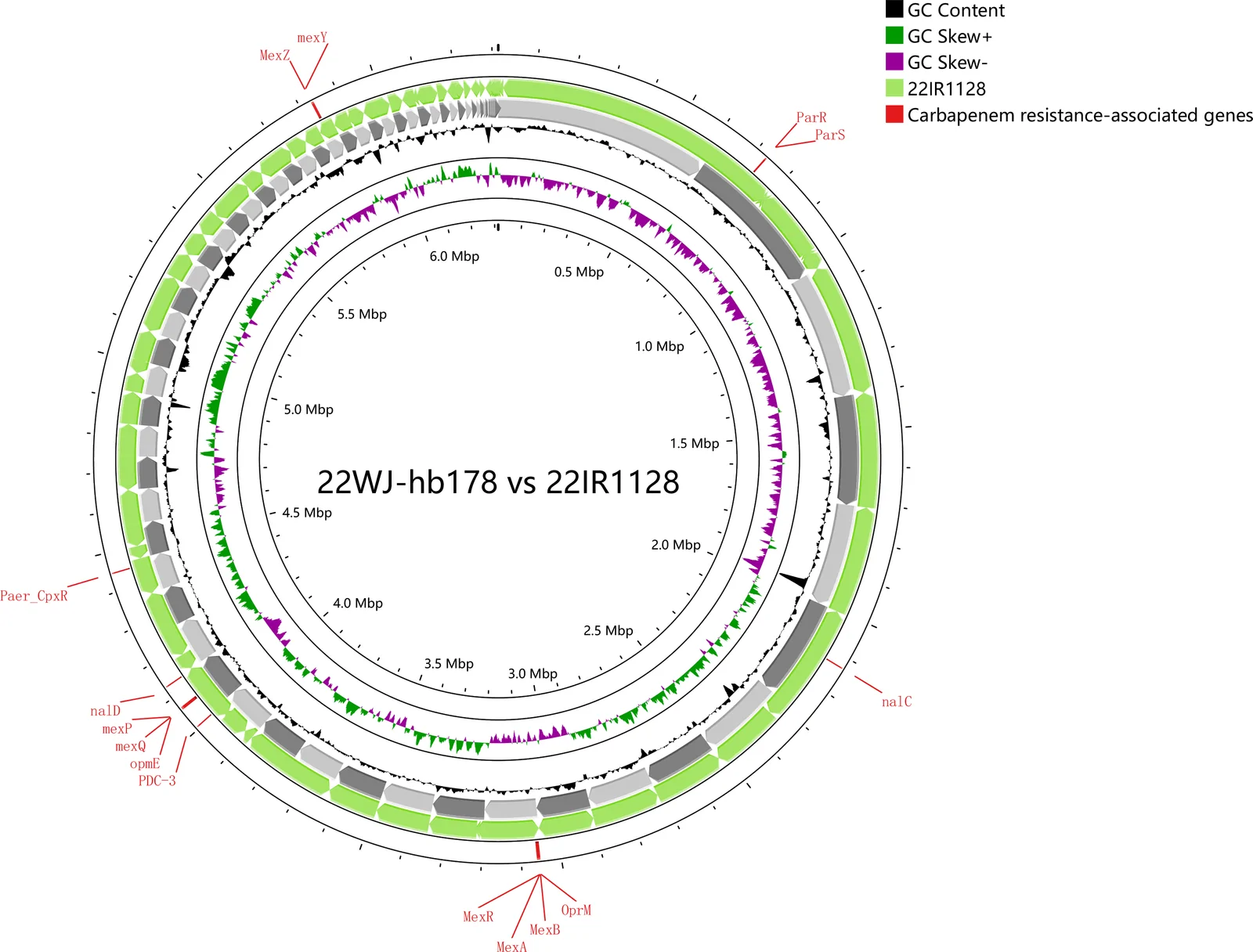
Circular genome comparison of *P. aeruginosa* strains 22WJ-hb178 (infectious isolate) and 22IR1128 (intestinal colonizing isolate). From innermost to outermost: the gray-white alternating ring represents the reference genome of strain 22WJ-hb178. The light green ring denotes the alignment coverage of strain 22IR1128, demonstrating near-complete genomic collinearity and high sequence identity. Inner tracks illustrate GC content (black), GC skew positive (green), and GC skew negative (purple). Key carbapenem resistance-associated genes and efflux pump regulators are annotated.

Strain 22WJ-hb178 was isolated from the blood specimen of a child and was identified as the pathogen causing bloodstream infection in the patient. KmerFinder 3.2 analysis confirmed that 22WJ-hb178 was *P. aeruginosa* with ST676. Strain 22IR1128 was an intestinal colonizing strain, which was also identified as *P. aeruginosa* with ST676 by KmerFinder 3.2. The two strains carried identical carbapenem resistance-associated genes, namely the MexPQ-OpmE multidrug efflux complex, the MexAB-OprM efflux complex, the MexXY-OprM efflux complex, the two-component sensor ParRS, and *PDC-3*. The ANI between the two strains was 99.9957%. Comparative genomic analysis of 22WJ-hb178 and 22IR1128 is shown in Fig. 5.

## DISCUSSION

CROs pose a serious threat to the prognosis of immunocompromised patients, especially pediatric patients with hematological malignancies who are at high risk of infection due to chemotherapy-induced myelosuppression and long-term hospitalization (32, 33). This study focused on CRO strains isolated from pediatric patients with hematological malignancies, systematically investigating their species distribution, AMR gene profiles, and genomic relationships between infectious and intestinal colonizing strains, aiming to provide basic data for clinical prevention and control of CRO infections in this specific population.

The clinical and demographic characteristics of the pediatric patients in this study reflect the typical profile of this high-risk population, with a predominance of certain underlying diseases (ALL/AML) and a high incidence of bloodstream infections. These characteristics provide a foundation for identifying high-risk groups for CRO infections, enabling clinicians to implement targeted prevention and control strategies tailored to this specific population. The species distribution observed among the infectious CRO strains aligns with previous reports from hematology wards, where gram-negative bacilli such as *P. aeruginosa*, *K. pneumoniae*, and *E. hormaechei* predominate (34, 35). Notably, we observed substantial species- and strain-level heterogeneity, with most isolates belonging to distinct sequence types. This suggests that CRO infections in this setting are driven by diverse clones rather than a single epidemic strain, which is consistent with the sporadic nature of CRO acquisition in immunocompromised hosts. Numerous drug resistance-related genes were detected in the CRO strains, with a subset being specifically associated with carbapenem resistance. Antibiotic efflux and antibiotic inactivation were the primary resistance mechanisms, consistent with the known resistance profiles of gram-negative bacilli (1, 36). Notably, distinct patterns of AMR gene carriage and resistance mechanisms were observed across different bacterial species, reflecting species-specific adaptations to carbapenem exposure. These species-specific characteristics may be linked to the intrinsic genetic background of each bacterial species and their ability to adapt to the clinical environment.

A subset of pediatric patients in this study harbored both infectious and intestinal colonizing CRO strains, and comparative genomic analysis revealed two distinct genomic relationship patterns between these paired strains. For two pairs of strains, high ANI and identical carbapenem resistance-associated genes indicated that the intestinal colonizing strains were identical to the corresponding infectious strains. This finding supports the hypothesis that the intestinal tract may serve as a reservoir for CROs, potentially contributing to subsequent infections in this high-risk population. In contrast, the remaining pair of strains belonged to different species despite sharing an identical carbapenemase gene and its associated genetic background. This observation implies potential horizontal transfer of carbapenemase genes between different bacterial species via mobile genetic elements, consistent with previous reports highlighting the role of transposons in carbapenemase gene dissemination (34). However, this conclusion is based on a single pair of strains, necessitating additional cases to verify the frequency of such horizontal gene transfer clinically.

It is important to acknowledge the limitations of this study. First, the sample size of this study was relatively small (19 CRO strains from 19 patients), which is mainly due to the narrow scope of the study population—pediatric patients with hematological malignancies are a special and small group, and the incidence of CRO infections in this population is relatively low, making it difficult to expand the sample size. Therefore, the findings of this study should be interpreted with caution, and the generalizability of the results may be limited. However, as a single-center study focusing on this specific high-risk population, the data obtained are still valuable for providing preliminary insights into CRO infections in pediatric patients with hematological malignancies. Second, this study only performed genomic analysis to predict AMR genes and their resistance mechanisms, and lacked functional verification (such as gene knockout or overexpression experiments) and phenotype-genotype association verification. Thus, the causal relationship between the detected AMR genes and carbapenem resistance phenotypes cannot be definitively confirmed, and the actual functional role of these genes in carbapenem resistance requires further validation through *in vitro* and *in vivo* experiments. Additionally, genomic relatedness between paired colonizing and infectious strains was assessed solely by average nucleotide identity based on short-read assemblies, without high-resolution core genome single nucleotide polymorphism analysis. We will address this limitation in future work using long-read sequencing to generate closed genomes and further dissect the within-host microevolution of CRO strains.

### Conclusions

In conclusion, this study systematically analyzed the species distribution, AMR gene profiles, and genomic relationships between infectious and intestinal colonizing CRO strains in pediatric patients with hematological malignancies. The findings highlight the predominant CRO species and key carbapenem resistance mechanisms in this population, as well as the potential role of the intestinal tract as a CRO reservoir and the possible horizontal transfer of carbapenemase genes. Despite the limitations of small sample size and lack of functional verification, this study provides valuable preliminary insights into CRO infections in this high-risk pediatric population. Future studies should aim to expand the sample size through multi-center collaboration and conduct functional experiments to further clarify the role of AMR genes in carbapenem resistance, thereby providing more robust evidence for the clinical prevention and control of CRO infections in pediatric patients with hematological malignancies.

## Data Availability

The sequence reads generated from all strains included in this study have been deposited in the GenBank database under the BioProject accession number PRJNA1455579.

## References

[1] Baleivanualala SC, Matanitobua S, Soqo V, Smita S, Limaono J, Sharma SC, Devi SV, Boseiwaqa LV, Vera N, Kumar S, Lalibuli A, Mailulu J, Wilson D, Samisoni Y, Crump JA, Ussher JE. 2024. Molecular and clinical epidemiology of carbapenem resistant *Acinetobacter baumannii*, *Pseudomonas aeruginosa* and Enterobacterales in Fiji: a multicentre prospective observational study. Lancet Reg Health West Pac 47:101095. doi:10.1016/j.lanwpc.2024.101095 PMC11166881 38867891

[2] Dong F, Lu J, Wang Y, Shi J, Zhen JH, Chu P, Zhen Y, Han SJ, Guo YL, Song WQ. 2017. A five-year surveillance of carbapenemase-producing *Klebsiella pneumoniae* in a pediatric hospital in China reveals increased predominance of NDM-1. Biomed Environ Sci 30:562–569. doi:10.3967/bes2017.075 28807096

[3] Trecarichi EM, Pagano L, Martino B, Candoni A, Di Blasi R, Nadali G, Fianchi L, Delia M, Sica S, Perriello V, Busca A, Aversa F, Fanci R, Melillo L, Lessi F, Del Principe MI, Cattaneo C, Tumbarello M, HaematologicMalignancies Associated Bloodstream Infections Surveillance (HEMABIS) registry - Sorveglianza Epidemiologica Infezioni Funginein Emopatie Maligne(SEIFEM) group, Italy. 2016. Bloodstream infections caused by *Klebsiella pneumoniae* in onco-hematological patients: clinical impact of carbapenem resistance in a multicentre prospective survey. Am J Hematol 91:1076–1081. doi:10.1002/ajh.24489 27428072

[4] Xiong Z, Zhang C, Sarbandi K, Liang Z, Mai J, Liang B, Cai H, Chen X, Gao F, Lan F, Liu X, Liu S, Zhou Z. 2023. Clinical and molecular epidemiology of carbapenem-resistant *Enterobacteriaceae* in pediatric inpatients in South China. Microbiol Spectr 11:e0283923. doi:10.1128/spectrum.02839-23 PMC10714942 37819092

[5] Chiotos K, Tamma PD, Flett KB, Naumann M, Karandikar MV, Bilker WB, Zaoutis T, Han JH. 2017. Multicenter study of the risk factors for colonization or infection with carbapenem-resistant *Enterobacteriaceae* in children. Antimicrob Agents Chemother 61:e01440-17. doi:10.1128/AAC.01440-17 PMC5700345 28971864

[6] Li J, Wu W, Wu M, Zhou Z, Wang J, Qiu M, Xu L, Ren J, Wu X. 2024. Clinical and molecular characteristics of patients with bloodstream infections caused by KPC and NDM co-producing carbapenem-resistant *Klebsiella pneumoniae*. Infect Drug Resist 17. doi:10.2147/IDR.S455146 PMC11073536 38711471

[7] Hu F, Pan Y, Li H, Han R, Liu X, Ma R, Wu Y, Lun H, Qin X, Li J, Wang A, Zhou M, Liu B, Zhou Z, He P. 2024. Carbapenem-resistant *Klebsiella pneumoniae* capsular types, antibiotic resistance and virulence factors in China: a longitudinal, multi-centre study. Nat Microbiol 9:814–829. doi:10.1038/s41564-024-01612-1 PMC10914598 38424289

[8] Richter SS, Marchaim D. 2017. Screening for carbapenem-resistant *Enterobacteriaceae*: who, when, and how? Virulence 8:417–426. doi:10.1080/21505594.2016.1255381 PMC5477693 27813699

[9] Zaidah AR, Mohammad NI, Suraiya S, Harun A. 2017. High burden of Carbapenem-resistant *Enterobacteriaceae* (CRE) fecal carriage at a teaching hospital: cost-effectiveness of screening in low-resource setting. Antimicrob Resist Infect Control 6:42. doi:10.1186/s13756-017-0200-5 PMC5414356 28473912

[10] Salomão MC, Freire MP, Boszczowski I, Raymundo SF, Guedes AR, Levin AS. 2020. Increased risk for carbapenem-resistant Enterobacteriaceae colonization in intensive care units after hospitalization in emergency department. Emerg Infect Dis 26:1156–1163. doi:10.3201/eid2606.190965 PMC7258474 32267827

[11] Queenan AM, Bush K. 2007. Carbapenemases: the versatile beta-lactamases. Clin Microbiol Rev 20:440–458, doi:10.1128/CMR.00001-07 PMC1932750 17630334

[12] Potter RF, D’Souza AW, Dantas G. 2016. The rapid spread of carbapenem-resistant *Enterobacteriaceae*. Drug Resist Updat 29:30–46. doi:10.1016/j.drup.2016.09.002 PMC5140036 27912842

[13] Little ML, Qin X, Zerr DM, Weissman SJ. 2012. Molecular diversity in mechanisms of carbapenem resistance in paediatric *Enterobacteriaceae*. Int J Antimicrob Agents 39:52–57. doi:10.1016/j.ijantimicag.2011.09.014 PMC3237943 22055532

[14] Acman M, Wang R, van Dorp L, Shaw LP, Wang Q, Luhmann N, Yin Y, Sun S, Chen H, Wang H, Balloux F. 2022. Role of mobile genetic elements in the global dissemination of the carbapenem resistance gene *bla*_NDM_. Nat Commun 13:1131. doi:10.1038/s41467-022-28819-2 PMC8894482 35241674

[15] Nigro SJ, Hall RM. 2016. Structure and context of *Acinetobacter* transposons carrying the *oxa23* carbapenemase gene. J Antimicrob Chemother 71:1135–1147. doi:10.1093/jac/dkv440 26755496

[16] Harmer CJ, Hall RM. 2024. IS26 and the IS26 family: versatile resistance gene movers and genome reorganizers. Microbiol Mol Biol Rev 88:e0011922. doi:10.1128/mmbr.00119-22 PMC11332343 38436262

[17] Gao H, Liu Y, Wang R, Wang Q, Jin L, Wang H. 2020. The transferability and evolution of NDM-1 and KPC-2 co-producing *Klebsiella pneumoniae* from clinical settings. EBioMedicine 51:102599. doi:10.1016/j.ebiom.2019.102599 PMC6948161 31911273

[18] Huang J, Zhang S, Zhao Z, Chen M, Cao Y, Li B. 2021. Acquisition of a stable and transferable *bla*_NDM-5_-positive plasmid with low fitness cost leading to ceftazidime/avibactam resistance in KPC-2-producing *Klebsiella pneumoniae* during treatment. Front Cell Infect Microbiol 11:658070. doi:10.3389/fcimb.2021.658070 PMC8329419 34354959

[19] Zhou Y, Wu X, Wu C, Zhou P, Yang Y, Wang B, Xu Y, Zhao H, Guo Y, Yu J, Yu F. 2024. Emergence of KPC-2 and NDM-5-coproducing hypervirulent carbapenem-resistant *Klebsiella pneumoniae* with high-risk sequence types ST11 and ST15. mSphere 9:e0061223. doi:10.1128/msphere.00612-23 PMC10826354 38193656

[20] Cai Y, Hoo GSR, Lee W, Tan BH, Yoong J, Teo Y-Y, Graves N, Lye D, Kwa AL. 2022. Estimating the economic cost of carbapenem resistant *Enterobacterales* healthcare associated infections in Singapore acute-care hospitals. PLoS Glob Public Health 2:e0001311. doi:10.1371/journal.pgph.0001311 PMC10021918 36962882

[21] Tamma PD, Aitken SL, Bonomo RA, Mathers AJ, van Duin D, Clancy CJ. 2023. Infectious diseases society of America 2023 guidance on the treatment of antimicrobial resistant gram-negative infections. Clin Infect Dis:ciad428. doi:10.1093/cid/ciad428 37463564

[22] Zeng M, Xia J, Zong Z, Shi Y, Ni Y, Hu F, Chen Y, Zhuo C, Hu B, Lv X, et al.. 2023. Guidelines for the diagnosis, treatment, prevention and control of infections caused by carbapenem-resistant gram-negative bacilli. J Microbiol Immunol Infect 56:653–671. doi:10.1016/j.jmii.2023.01.017 36868960

[23] Performance standards for antimicrobial susceptibility testing. 2025. 35th ed. CLSI Supplement M100. Clinical and Laboratory Standards Institute.

[24] Chen S, Zhou Y, Chen Y, Gu J. 2018. fastp: an ultra-fast all-in-one FASTQ preprocessor. Bioinformatics 34:i884–i890. doi:10.1093/bioinformatics/bty560 PMC6129281 30423086

[25] Wick RR, Judd LM, Gorrie CL, Holt KE. 2017. Unicycler: resolving bacterial genome assemblies from short and long sequencing reads. PLoS Comput Biol 13:e1005595. doi:10.1371/journal.pcbi.1005595 PMC5481147 28594827

[26] Chklovski A, Parks DH, Woodcroft BJ, Tyson GW. 2023. CheckM2: a rapid, scalable and accurate tool for assessing microbial genome quality using machine learning. Nat Methods 20:1203–1212. doi:10.1038/s41592-023-01940-w 37500759

[27] Seemann T. 2014. Prokka: rapid prokaryotic genome annotation. Bioinformatics 30:2068–2069. doi:10.1093/bioinformatics/btu153 24642063

[28] Jain C, Rodriguez-R LM, Phillippy AM, Konstantinidis KT, Aluru S. 2018. High throughput ANI analysis of 90K prokaryotic genomes reveals clear species boundaries. Nat Commun 9:5114. doi:10.1038/s41467-018-07641-9 PMC6269478 30504855

[29] Viver T, Conrad RE, Rodriguez-R LM, Ramírez AS, Venter SN, Rocha-Cárdenas J, Llabrés M, Amann R, Konstantinidis KT, Rossello-Mora R. 2024. Towards estimating the number of strains that make up a natural bacterial population. Nat Commun 15:544. doi:10.1038/s41467-023-44622-z PMC10791622 38228587

[30] Siguier P, Perochon J, Lestrade L, Mahillon J, Chandler M. 2006. ISfinder: the reference centre for bacterial insertion sequences. Nucleic Acids Res 34:D32–D36. doi:10.1093/nar/gkj014 PMC1347377 16381877

[31] Kolde R. 2019. Package “pheatmap”. R package;1(10)

[32] Ruhnke M, Arnold R, Gastmeier P. 2014. Infection control issues in patients with haematological malignancies in the era of multidrug-resistant bacteria. Lancet Oncol 15:e606–e619. doi:10.1016/S1470-2045(14)70344-4 25456379

[33] Zhou J, Sun J, Lu S, Han X, He J, Zhang P, Hu H, Zhang Y, Wang Y, Yang Q, Ji S, Zhou Z, Hua X, Wu X, Jiang Y, Du X, Yu Y. 2024. Clinical characteristics and prognosis of bloodstream infections with carbapenem-resistant gram-negative organisms in patients with hematological malignancies: a multicenter case-control study in China. J Infect 89:106331. doi:10.1016/j.jinf.2024.106331 39490814

[34] Kocer K, Boutin S, Dalpke AH, Heeg K, Mutters NT, Nurjadi D. 2020. Comparative genomic analysis reveals a high prevalence of inter-species *in vivo* transfer of carbapenem-resistance plasmids in patients with haematological malignancies. Clin Microbiol Infect 26:780. doi:10.1016/j.cmi.2019.10.014 31654794

[35] Lee J-Y, Kang C-I, Ko J-H, Lee WJ, Seok H-R, Park GE, Cho SY, Ha YE, Chung DR, Lee NY, Peck KR, Song J-H. 2016. Clinical features and risk factors for development of breakthrough gram-negative bacteremia during carbapenem therapy. Antimicrob Agents Chemother 60:6673–6678. doi:10.1128/AAC.00984-16 PMC5075072 27572416

[36] Tenover FC, Nicolau DP, Gill CM. 2022. Carbapenemase-producing *Pseudomonas aeruginosa* –an emerging challenge. Emerg Microbes Infect 11:811–814. doi:10.1080/22221751.2022.2048972 PMC8920394 35240944

